# Linear B-spline finite element method for the generalized diffusion equation with delay

**DOI:** 10.1186/s13104-022-06078-0

**Published:** 2022-06-03

**Authors:** Gemeda Tolessa Lubo, Gemechis File Duressa

**Affiliations:** 1 Department of Mathematics, Wollega University, Nekemte, Ethiopia; 2 Department of Mathematics, Jimma University, Jimma, Ethiopia

**Keywords:** Generalized diffusion equation with delay, Finite element, Linear B-spline, 65M30, 65M60

## Abstract

**Objectives:**

The main aim of this paper is to develop a linear B-spline finite element method for solving generalized diffusion equations with delay. The linear B-spline basis function is used to discretize the space variable. The time discretization process is based on Crank-Nicolson. The benefit of the scheme is that the numerical solution is obtained as a smooth piecewise continuous function which empowers one to find an approximate solution at any desired position in the domain.

**Result:**

Sufficient and necessary conditions for the numerical method to be asymptotically stable are derived. The convergence of the numerical method is studied. Some numerical experiments are performed to verify the applicability of the numerical method.

## Introduction

In this paper, we consider a class of the generalized delay diffusion equation of the form

1 $$\begin{aligned} \left\{ \begin{array}{lll} \frac{\partial u(x,t)}{\partial t} = a_{1}\frac{\partial ^{2} u(x,t)}{\partial x^{2}}+a_{2}\frac{\partial ^{2} u(x,t-\tau )}{\partial x^{2}} ,t>0,0< x <\pi , \\ u(x,t) =\psi \ (x,t), ~~-\tau \le t\le 0,0 \le x \le \pi , \\ u(0,t)=u(\pi ,t)=0, t>0, \end{array} \right. \end{aligned}$$

with $a_{1},a_{2} \in {\mathbb {R}}$ are real numbers and $\tau >0$ is a delay constant. The delay diffusion equation has several applications in science and engineering [1–5]. The generalized delay diffusion equation has intrinsic complex nature because its exact solutions are difficult to obtain. Therefore, one has to mostly rely on numerical treatments. Jackiewicz and Zubik-Kowal [6] used spectral collocation and waveform relaxation methods to investigate nonlinear partial differential equations with delay. Chen and Wang [7] used the variational iteration method to study a neutral functional differential equation with delays. The numerical treatments of the generalized delay diffusion equations were studied by many authors(see for instance [8–11]). Test equation of the type Eq. (1) is also considered in [12, 13]. In these works, the authors applied the separation of the variables to solve analytically.

The finite element method (FEM) is a well-established numerical method for solving partial differential equations (PDEs). The method approximates the exact solution by using piecewise polynomials or B-spline basis functions. B-splines as finite element basis functions provide the required continuity and smoothness. The use of various degrees of B-spline functions to obtain the numerical solutions of some PDEs has been shown to provide easy and simple algorithms. For instance, B-spline finite elements have been widely applied to solve elliptic equations [14, 15], Korteweg-De Vries equation [16–18], Burgers’ equation [19–22], regularized long-wave equation [23, 24], Fokker-Planck equation [25], advection-diffusion equation [26], and generalized equal width wave equation [27], etc., successfully. However, to the best knowledge of the authors, the B-spline FEM method is not considered for finding the approximate solution of the diffusion equation with delay. In this paper, we have applied a linear B-spline FEM to find numerical solutions to the problem under consideration.

### Notations

Let $H^{r}=H^{r}(\Omega )= W_2^r(\Omega )$ denotes the Sobolev spaces of order *r* with respective to norm $\left\| . \right\| _{r}$ defined as

$$\begin{aligned} \quad \left\| \nu \right\| =\quad \left\| \nu \right\| _{L_{2}}:=\Bigg (\int _{\Omega } \nu (x)^{2}dx\Bigg )^{\frac{1}{2}} \end{aligned}$$

and

$$\begin{aligned} \quad \left\| \nu \right\| _{r} =\quad \left\| \nu \right\| _{H^{r}}:=\Bigg (\sum _{i \le r}\quad \left\| \frac{\partial ^{i} \nu (x)}{\partial x^{i}}\right\| ^{2}\Bigg )^{\frac{1}{2}}. \end{aligned}$$

Let $\nu (x), w(x)(x\in \Omega )$ be real valued functions.

$$\begin{aligned} (\nu (x),w(x)):=\int _{\Omega } \nu (x) w(x)dx,~~~~~~ (\nabla \nu (x),\nabla w(x)) := \int _{\Omega }\frac{\partial \nu (x)}{\partial x}\frac{\partial w(x)}{\partial x}dx. \end{aligned}$$

### Assumption

Assume $$u(t):=u(.,t), u_{t}(t):=u_{t}(.,t),u_{tt}(t):=u_{tt}(.,t), u_{ttt}(t):=u_{ttt}(.,t),\psi (t):=\psi \ (.,t)$$, and $$\psi _{t} (t):=\psi _{t}(.,t)$$.

## Main text

## Description of the method

Let $\Delta t = \tau /m$ be a step size with $m \ge 1$, the grid points $t_{n} = n\Delta t (n=0,1,\dots )$ and be the approximation in $S_h$ of *u*(*t*) at $t=t_{n} = n\Delta t$. We partition the *x* -axis into *N* finite element by choosing a set of equally-spaced knots $\{x_{k}\}_{k=0}^{N}$ such at $0 = x_{0}<x_{1}<\dots x_{N-1}< x_{N}=\pi$ and $x_{i+1}-x_{i}=h, i =0,1,2, \dots ,N -1$.

The linear B-spline basis functions is chosen as follows:

2 $$\begin{aligned} Q_{j}(x) = \left\{ \begin{array}{lll} \frac{x-x_{j-1}}{x_{j}-x_{j-1}}, x \in [x_{j-1},x_{j}]\\ \frac{x_{j+1}-x}{x_{j+1}-x_{j}}, x \in [x_{j},x_{j+1}] \\ 0,\,\,\,\,\,\,\,\,\,\,\,\,\,\,\,\,\,x \notin [x_{j-1},x_{j+1}]\\ \end{array}\right. j =1,2,\dots ,N-1. \end{aligned}$$

Applying Green’s formula to the second and third terms of equation (1) we obtain

3 $$\begin{aligned} (u_{t}(x,t),\nu )+a_{1}(\nabla u(x,t),\nabla \nu )+a_{2}( \nabla u(x,t-\tau ),\nabla \nu )=0,\forall \nu \in H_0^1(\Omega ),t >0. \end{aligned}$$

Define the space

$$\begin{aligned} S_{h} =\{\zeta :\zeta \in C^{2}([0,\pi ]),\zeta |_{[x_{n-1},x_{n}]} \in P^{1} ,1\le n\le N,\zeta (0) = \zeta (\pi ) = 0 \}, \end{aligned}$$

where $P^{1}$ is the space of all polynomials degree less or equal to 1.

We can find the approximate solution $u_{h}(t): =u_{h}(.,t)$ belonging to $S_{h}$ for each *t*, so that

4 $$\begin{aligned} \left\{ \begin{array}{lll} (u_{h,t}(t,\zeta ))+ a_{1}(\nabla u_{h}(t),\nabla \zeta )+a_{2}( \nabla u_{h}(t-\tau ),\nabla \zeta )=0,\forall \zeta \in S_{h},t>0,\\ u_{h}(x,t)=\psi _{h}(x,t)=0, t>0, \end{array} \right. \end{aligned}$$

where $\psi _{h}(.,t)$ is an approximation of $\psi (.,t)$ in $S_{h}$.

Let $\Delta t =\tau / m$ be a given step size with $m \ge 1$, the grid points $t_{n} = n\Delta t ( n=0,1,\dots )$ and $U^{n}$ be the approximation in $S_{h}$ of *u*(*t*) at $t =t_{n} =n\Delta t$.

Application of Galerkin Crank-Nicloson method to Eq. (4) gives a numerical scheme of the following type

5 $$\begin{aligned} \Bigg (\frac{U^{n}-U^{n-1}}{\Delta t},\zeta \Bigg ) +a_{1} \Bigg (\frac{\nabla U^{n}+\nabla U^{n-1}}{2},\nabla \zeta \Bigg ) +a_{2}\Bigg (\frac{\nabla U^{n-m}+\nabla U^{n-m-1}}{2 },\nabla \zeta \Bigg )=0, \end{aligned}$$

where $$U^{n}(.) = \psi (.,t_{n})$$ for $$-m \le n \le 0$$.

Let

6 $$\begin{aligned} U^{n}(x) :=\sum _{j=1}^{N-1}Q_{j}(x) \alpha _j^{n}. \end{aligned}$$

Substituting Eq. (6) into Eq. (5) and choosing $\zeta =Q_{i},i=0,\dots ,N-1$, we get

$$\begin{aligned} \frac{1}{\Delta t}\sum _{j=1}^{N-1}(\alpha _j^{n}-\alpha _j^{n-1})(Q_{i}(x),Q_{j}(x))=-\frac{a_{1}}{2}\sum _{j=1}^{N-1}(\alpha _j^{n}+\alpha _j^{n-1})(\nabla Q_{i}(x),\nabla Q_{j}(x)) \end{aligned}$$

7 $$\begin{aligned} -\frac{a_{2}}{2}\sum _{j=1}^{N-1}(\alpha _j^{n-m}+\alpha _j^{n-m-1})(\nabla Q_{i}(x),\nabla Q_{j}(x)), \end{aligned}$$

which can be rewritten as

8 $$\begin{aligned} \frac{1}{\Delta t}\sum _{j=1}^{N-1}(\alpha _j^{n}-\alpha _j^{n-1})\int _{0}^{\pi }Q_{i}(x)Q_{j}(x)dx =-\frac{a_{1}}{2}\sum _{j=1}^{N-1}(\alpha _j^{n}+\alpha _j^{n-1})\int _{0}^{\pi }Q_{i}'(x)Q_{j}'(x)dx \\ \quad -\frac{a_{2}}{2}\sum _{j=1}^{N-1}(\alpha _j^{n-m}+\alpha _j^{n-m-1})\int _{0}^{\pi }Q_{i}'(x)Q_{j}'(x)dx. \end{aligned}$$

Define the following matrices:

9 $$A= (a_{i,j})_{i,j=1}^{N-1}=\int _{0}^{\pi }Q_{i}'(x)Q_{j}'(x)dx,$$

10 $$B= (b_{i,j})_{i,j=1}^{N-1}=\int _{0}^{\pi }Q_{i}(x)Q_{j}(x)dx.$$

The $(N -1)\times (N-1)$ matrices *A* and *B* are given as follows

11 $$\begin{aligned} A= & {} \frac{1}{h}\left( \begin{array}{cccccc} 2 &{} -1 &{} 0 &{}\ldots &{} 0 &{} 0 \\ -1 &{} 2 &{} -1 &{}\ldots &{} 0 &{} 0 \\ 0 &{} -1 &{} 2 &{}\ldots &{} 0 &{} 0 \\ \vdots &{}\vdots &{}\vdots &{}\ddots &{}\vdots &{} \vdots \\ 0 &{} 0 &{} 0 &{}\ldots &{} 2 &{} -1 \\ 0 &{} 0 &{} 0 &{}\ldots &{} -1 &{} 2 \\ \end{array} \right) \nonumber \\ B= & {} \frac{h}{6}\left( \begin{array}{cccccc} 4 &{} 1 &{} 0 &{}\ldots &{} 0 &{} 0 \\ 1 &{} 4 &{} 1 &{}\ldots &{} 0 &{} 0 \\ 0 &{} 1 &{} 4 &{}\ldots &{} 0 &{} 0 \\ \vdots &{}\vdots &{}\vdots &{}\ddots &{}\vdots &{} \vdots \\ 0 &{} 0 &{} 0 &{}\ldots &{} 4 &{} 1 \\ 0 &{} 0 &{} 0 &{}\ldots &{} 1 &{} 4 \\ \end{array} \right) \nonumber \\&\left\{ \begin{array}{lll} (B +\frac{1}{2}a_{1}\Delta t A)\alpha ^{n} =(B -\frac{1}{2}a_{1}\Delta t A )\alpha ^{n-1} -\frac{1}{2}a_{2}\Delta t A(\alpha ^{n-m} + \alpha ^{n-m-1}),\\ \alpha ^{n} = \gamma ^{n} ,for -m \le n \le 0. \end{array}\right. \end{aligned}$$

with $\gamma ^{n}=\psi (t_{n})$ an initial approximation and $\alpha ^{n}:=(\alpha _1,\dots ,\alpha _{N} )^{T}$, and $B +\frac{1}{2}a_{1}\Delta t A$ is positive definite and hence, in particular, invertible. Therefore, it has a unique solution.

## Stability analysis

### Definition 1

If the solution $U^{n}$ of Eq. (5) corresponding to any sufficiently differentiable function $\psi _{h}(x,t)$ with $\psi _{h}(0,t)$ =$\psi _{h}(\pi ,t)$ satisfies

12 $$\begin{aligned} \lim _{n\rightarrow \infty }{U}^{n} = 0,x \in [0,1], \end{aligned}$$

then the zero solution of Eq. (5) is called asymptotically stable.

Let $K :=[x_{i},x_{i+1}]$ be an element the finite element, and ${\tilde{K}}:=[-1,1]$ be the reference element in $\eta$ -plane. Then

$$\begin{aligned} \int _{K}{\tilde{Q}}_{i}{\tilde{Q}}_{j}dx=\frac{h}{2}\int _{{\tilde{K}}}\tilde{\tilde{Q_{i}}}\tilde{\tilde{Q_{j}}}d\eta , \int _{K}\nabla \tilde{ Q_{i}}\nabla \tilde{Q_{j}}dx=\frac{2}{h}\int _{{\tilde{K}}}\nabla \tilde{\tilde{Q_{i}}}\nabla \tilde{\tilde{Q_{j}}}d\eta , \end{aligned}$$

where $${\tilde{B}}=\int _{{\tilde{K}}}\tilde{\tilde{Q_{i}}}\tilde{\tilde{Q_{j}}}d\eta$$ and $${\tilde{A}} = \int _{{\tilde{K}}}\nabla \tilde{\tilde{Q_{i}}}\nabla \tilde{\tilde{Q_{j}}}d\eta$$.

From Eq. (8),

13 $$\begin{aligned} \alpha ^{n}= & {} \Bigg (\frac{h}{2}{\tilde{B}} +\frac{a_{1} \Delta t}{h}{\tilde{A}} \Bigg )^{-1}\Bigg (\frac{h}{2}{\tilde{B}} -\frac{a_{1}\Delta t}{h}{\tilde{A}} \Bigg )\alpha ^{n-1} \nonumber \\&-\frac{a_{2}\Delta t}{h}\Bigg (\frac{h}{2}{\tilde{B}} +\frac{a_{1}\Delta t}{h}{\tilde{A}} \Bigg )^{-1}{\tilde{A}}(\alpha ^{n-m}+\alpha ^{n-m-1}) \end{aligned}$$

14 $$\begin{aligned} \alpha ^{n}= & {} \Bigg (I +\frac{2a_{1}\Delta t}{h^{2}}{\tilde{B}}^{-1}{\tilde{A}}\Bigg )^{-1}\Bigg (I -\frac{2a_{1}\Delta t}{h^{2}}{\tilde{B}}^{-1}{\tilde{A}}\Bigg )\alpha ^{n-1} \nonumber \\&-\frac{2a_{2}\Delta t}{h^{2}}\Bigg (I +\frac{2a_{1}\Delta t}{h^{2}}{\tilde{B}}^{-1}{\tilde{A}} \Bigg )^{-1}{\tilde{B}}^{-1}{\tilde{A}}(\alpha ^{n-m}+\alpha ^{n-m-1}). \end{aligned}$$

Let $\alpha ^{n} =\gamma ^{n}C_{1}$, where $C_{1}$ is a constant vector. The characteristic of Eq. (14) is:

15 $$\begin{aligned} \gamma ^{m} -\Bigg (\frac{1 -\frac{2a_{1}\Delta t}{h^{2}}\lambda _{{\tilde{B}}^{-1}{\tilde{A}}}}{1 +\frac{2a_{1}\Delta t}{h^{2}}\lambda _{{\tilde{B}}^{-1}{\tilde{A}}}}\Bigg )\gamma ^{m-1}- \Bigg (\frac{\frac{2a_{2}\Delta t}{h^{2}}\lambda _{{\tilde{B}}^{-1}{\tilde{A}}}}{1 +\frac{2a_{1}\Delta t}{h^{2}}\lambda _{{\tilde{B}}^{-1}{\tilde{A}}}}\Bigg ) (\gamma +1)=0, \end{aligned}$$

where $\gamma _{{\tilde{B}}^{-1}{\tilde{A}}}$ denotes the corresponding eigenvalue of ${\tilde{B}}^{-1}{\tilde{A}}$.

### Lemma 1

[28] Let $\kappa _{m}(z) =\alpha (z)z^{m} -\beta (z)$ be a polynomial, with $\alpha (z)$ and $\beta (z)$ are polynomials of zero degree. Then $\kappa _{m}(z)$ is a Schur polynomial for $m \ge 1$ if and only if the following conditions hold

(i) $$\alpha (z) =0 \Rightarrow \left| z\right| < 1,$$
(ii) $$\left| \beta (z)\right| \le \left| \alpha (z)\right| ,\forall z \in {\mathbb {C}} , \left| z\right| = 1,$$ and
(iii) $$\kappa _{m}(z) \ne 0 ,\forall z\in {\mathbb {C}}, \left| z\right| = 1.$$

### Theorem 1

Suppose that $0 \le a_{2} <a_{1}$. Then the zero solution of the B-spline finite element method is delay-independently asymptotically stable.

### Proof

Let $$\alpha {(\gamma )} =\gamma -\frac{1 -\frac{2a_{1} \Delta t}{h^{2}}\gamma _{{\tilde{B}}^{-1}{\tilde{A}}}}{1 +\frac{2a_{1} \Delta t}{h^{2}}\gamma _{{\tilde{B}}^{-1}{\tilde{A}}}}$$ and $$\beta {(\gamma })=\frac{\frac{2a_{2} \Delta t}{h^{2}}\gamma _{{\tilde{B}}^{-1}{\tilde{A}}}}{1 +\frac{2a_{1} \Delta t}{h^{2}}\gamma _{{\tilde{B}}^{-1}{\tilde{A}}}}(\gamma +1)$$.

(i) If $$\alpha {(\gamma )}=0$$, then $$\left| \gamma \right| =\left| \frac{1 -\frac{2a_{1} \Delta t}{h^{2}}\gamma _{{\tilde{B}}^{-1}{\tilde{A}}}}{1 +\frac{2a_{1} \Delta t}{h^{2}}\gamma _{{\tilde{B}}^{-1}{\tilde{A}}}}\right| < 1.$$

(ii) For $\forall \gamma \in {\mathbb {C}}$, $\left| \gamma \right| =1$, represent $\gamma =\cos \varrho +i\sin \varrho$, then we get

$$\begin{aligned} \frac{\gamma -1}{\gamma +1}=\frac{\cos \varrho -1 +i\sin \varrho }{\cos \varrho +1 +i\sin \varrho } =\frac{2i\sin \varrho }{2+2\cos \varrho }. \end{aligned}$$

We obtain

$$\begin{aligned}&\left| \frac{\alpha (\gamma ) }{\gamma +1}\right| =\left| \frac{\gamma -\frac{1 -\frac{2a_{1} \Delta t}{h^{2}}\gamma _{{\tilde{B}}^{-1}{\tilde{A}}}}{1 +\frac{2a_{1} \Delta t}{h^{2}}\gamma _{{\tilde{B}}^{-1}{\tilde{A}}}}}{\gamma +1} \right| =\left| \frac{(\gamma -1)}{(\gamma +1)(1+\frac{2a_{1}\Delta t}{h^{2}}\gamma _{{\tilde{B}}^{-1}{\tilde{A}}})}+\frac{\frac{2a_{1}\Delta t}{h^{2}}\gamma _{{\tilde{B}}^{-1}{\tilde{A}}}}{1+\frac{2a_{1} \Delta t}{h^{2}}\gamma _{{\tilde{B}}^{-1}{\tilde{A}}}}\right| \\&\quad \ge \frac{\frac{2a_{1} \Delta t}{h^{2}}\gamma _{{\tilde{B}}^{-1}{\tilde{A}}}}{1+\frac{2a_{1} \Delta t}{h^{2}}\gamma _{{\tilde{B}}^{-1}{\tilde{A}}}}>\frac{\frac{2a_{2} \Delta t}{h^{2}}\gamma _{{\tilde{B}}^{-1}{\tilde{A}}}}{1+\frac{2a_{1} \Delta t}{h^{2}}\gamma _{{\tilde{B}}^{-1}{\tilde{A}}}}=\left| \frac{\beta (\gamma ) }{\gamma +1}\right| . \end{aligned}$$

(iii) By (ii), it is straightforward. $\square$

## Convergence Analysis

In this section, we present the convergence analysis for the proposed method.

The Ritz projection $R_{h}:H_0^{1}(\Omega ) \rightarrow S_{h}$ is a mapping for any $\nu \in H_0^{1}(\Omega )$ such that

16 $$\begin{aligned} (\nabla R_{h}\nu -\nu ,\nabla w)=0, \forall w \in S_{h}. \end{aligned}$$

### Lemma 2

Assume that for any $v \in H^{s}(\Omega )\cap H_0^{1}(\Omega )$,

$$\begin{aligned} \inf _{\zeta \in {S}_{h}}\{\left\| \nu -\zeta \right\| + h\left\| \nabla (\nu -\zeta )\right\| \} \le Ch^{s}\left\| \nu \right\| _{s},for ~~ 1\le s \le r . \end{aligned}$$

holds. Then, with $R_{h}$ defined by Eq. (16), we have

$$\begin{aligned} \left\| R_{h}\nu -\nu \right\| +h\left\| \nabla (R_{h}\nu -\nu )\right\| \le Ch^{s}\left\| \nu \right\| _{s}, for~~ any ~ \nu ~ \in H^{s}(\Omega )\cap H_0^{1}(\Omega ), 1\le s \le r. \end{aligned}$$

The number *r* is referred to as the order of accuracy of the family $\{S_{h}\}$. For the case of piecewise linear B-spline basis function, $r =2$.

Define $u(t):= u(.,t)$ and $u:[0,+\infty ) \rightarrow H_0^{1}(\Omega )$. Let $D_{h}: H_0^{1}(\Omega )\rightarrow S_h$ by

17 $$\begin{aligned} a_{1}(\nabla D_{h}u(t) -\nabla u(t), \nabla \zeta ) +a_{2} ( \nabla D_{h} u(t-\tau )-\nabla u(t-\tau ),\nabla \zeta ) =0, \forall \zeta \in S_h \end{aligned}$$

and

18 $$\begin{aligned} D_{h}u(t)=R_{h}u(t)=R_{h}\psi (t),for ~ -\tau \le t \le 0. \end{aligned}$$

### Theorem 2

Let *u* and $U^{n}$ be the solution of (3) and (5), respectively. Assume that $\left\| u(t) -R_{h} u (t)\right\| \le Ch^{2} \left\| u (t)\right\| _{2}$, $\left\| u_{t}(t) -R_{h} u_{t} (t)\right\| \le Ch^{2} \left\| u _{t}(t)\right\| _{2}$, $-\tau \le t\le 0$ and $\left\| \psi _{h}(t)-\psi (t)\right\| \le Ch^{2}$, then

$$\begin{aligned} \left\| U^{n} -u (t_{n})\right\| \le C(h^{2}+(\Delta t)^{2}) ,for~~ n=1,2,... \end{aligned}$$

where *C* is a positive constant independent of *h* and $\Delta t$.

### Proof

Define

$$\begin{aligned} e^{n}=U^{n} -u (t_{n})=(U^{n} -D_{h}u (t_{n}))+(D_{h}u (t_{n})- u (t_{n} )) =\mu ^{n}+\sigma ^{n}, \end{aligned}$$

where

$$\mu ^{n}=U^{n} -D_{h}u (t_{n})$$, $$\sigma ^{n} = D_{h}u (t_{n})- u (t_{n} )$$, so that

$$\begin{aligned} \left\| U^{n} -u (t_{n})\right\| \le \left\| \mu ^{n}\right\| +\left\| \sigma ^{n}\right\| . \end{aligned}$$

The term $\sigma ^{n}(t) =\sigma (t_{n})$ is easily bounded by lemma 2.

19 $$\begin{aligned}&\Bigg (\frac{\mu ^{n}-\mu ^{n-1}}{\Delta t},\zeta \Bigg )+a_{1}\Bigg (\frac{\nabla \mu ^{n}+\nabla \mu ^{n-1}}{2},\nabla \zeta \Bigg )+a_{2}\Bigg (\frac{\nabla \mu ^{n-m}+\nabla \mu ^{n-m-1}}{2},\nabla \zeta \Bigg ) \nonumber \\&\quad =-(W^{n},\zeta ), \forall \zeta \in S_h, \end{aligned}$$

where

$$\begin{aligned} W^{n}= & {} \frac{D_{h} u(t_{n})-D_{h} u(t_{n-1})}{\Delta t} -\frac{ u_{t}(t_{n})+ u_{t}(t_{n-1})}{2} \\= & {} (D_{h}-I){\bar{\partial }} u(t_{n}) +\Bigg ({\bar{\partial }} u(t_{n})-\frac{u_{t}(t_{n})+u_{t}(t_{n-1})}{2}\Bigg )=:W_1^{n}+W_2^{n}. \end{aligned}$$

Setting $$\zeta =\frac{\mu ^{n}+\mu ^{n-1}}{2}$$, gives

$$\begin{aligned}&\Bigg ( \frac{\mu ^{n}-\mu ^{n-1}}{\Delta t},\frac{\mu ^{n}+\mu ^{n-1}}{2}\Bigg )+a_{1} \left\| \frac{\mu ^{n}+\mu ^{n-1}}{2} \right\| _1^{2} +a_{2}\Bigg (\frac{\nabla \mu ^{n-m}+\nabla \mu ^{n-m-1}}{2},\frac{\nabla \mu ^{n}+\nabla \mu ^{n-1}}{2}\Bigg ) \\&\quad =-\Bigg (W^{n},\frac{\mu ^{n}+\mu ^{n-1}}{2}\Bigg ). \end{aligned}$$

By applying Schwartz inequality,

$$\begin{aligned}&\Bigg ( \frac{\mu ^{n}-\mu ^{n-1}}{\Delta t},\frac{\mu ^{n}+\mu ^{n-1}}{2}\Bigg )+ \left\| \frac{\mu ^{n}+\mu ^{n-1}}{2} \right\| _1^{2} \le C\Bigg ( \left\| \frac{\mu ^{n-m}+\mu ^{n-m-1}}{2} \right\| _1^{2} \nonumber \\&\qquad +\left\| W^{n}\right\| \left\| \frac{\mu ^{n}+\mu ^{n-1}}{2} \right\| \Bigg ). \end{aligned}$$

So

$$\begin{aligned} \left\| \mu ^{n} \right\| ^{2} +\Delta t \left\| \frac{\mu ^{n}+\mu ^{n-1}}{2} \right\| _1^{2}\le C\Bigg (\left\| \mu ^{n-1} \right\| ^{2} +\Delta t \left\| \frac{\mu ^{n-m}+\mu ^{n-m-1}}{2} \right\| _1^{2} +(\Delta t )^{2}\left\| W^{n}\right\| ^{2}\Bigg ). \end{aligned}$$

We can assume that $n \in ((k-1)m,km],k \in N$. Then

$$\begin{aligned}&\Delta t \left\| \frac{\mu ^{n}+\mu ^{n-1}}{2} \right\| _1^{2}\le C\Bigg (\left\| \mu ^{n-1} \right\| ^{2} +\Delta t \left\| \frac{\mu ^{n-m}+\mu ^{n-m-1}}{2} \right\| _1^{2} +(\Delta t )^{2}\left\| W^{n}\right\| ^{2}\Bigg ) \\&\quad \le C\Bigg (\left\| \mu ^{n-1} \right\| ^{2} + \left\| \mu ^{n-m-1} \right\| ^{2}+\Delta t \left\| \frac{\mu ^{n-2m}+\mu ^{n-2m-1}}{2} \right\| _1^{2}+(\Delta t )^{2}( \left\| W^{n}\right\| ^{2}+\left\| W^{n-m}\right\| ^{2})\Bigg ) \\&\quad \le ...\le C\Bigg (\sum _{i=0}^{k-1}\left\| \mu ^{n-im-1} \right\| ^{2}+\Delta t \left\| \frac{\mu ^{n-km}+\mu ^{n-km-1}}{2} \right\| _1^{2}+(\Delta t )^{2} \sum _{i=0}^{k-1}\left\| W^{n-im}\right\| ^{2}\Bigg ). \end{aligned}$$

Therefore

$$\begin{aligned} \left\| \mu ^{n} \right\| ^{2}\le C\Bigg ( \sum _{i=0}^{k-1}\left\| \mu ^{n-im-1} \right\| ^{2}+\Delta t \left\| \frac{\mu ^{n-km}+\mu ^{n-km-1}}{2}\right\| _1^{2}+(\Delta t )^{2} \sum _{i=0}^{k-1}\left\| W^{n-im}\right\| ^{2}\Bigg ). \end{aligned}$$

By applying Gronwall inequality,

20 $$\begin{aligned} \left\| \mu ^{n} \right\| ^{2}\le C\Bigg ( \left\| \mu ^{0} \right\| ^{2}+\Delta t \left\| \frac{\mu ^{n-km}+\mu ^{n-km-1}}{2} \right\| _1^{2}+(\Delta t )^{2} \sum _{i=0}^{k-1}\left\| W ^{n-im}\right\| ^{2}\Bigg ). \end{aligned}$$

Write

$$\begin{aligned} W_1^{n} =(D_{h}-I){\tilde{\partial }}u(t_{n}) = \Delta t ^{-1}\int _{t_{n-1}}^{t_{n}}(D_{h}-I)u_{t}(t)dt, \end{aligned}$$

so

21 $$\begin{aligned} (\Delta t )^{2} \sum _{i=1}^{k-1}\left\| W_1^{n-im}\right\| ^{2}\le \sum _{i=1}^{k-1}\Bigg (\int _{t_{n -im-1}}^{t_{n-im}} Ch^{2}\left\| u_{t}(t) \right\| _{2} dt\Bigg )^{2} \le Ch^{2(2)}. \end{aligned}$$

Further

$$\begin{aligned} \left\| \Delta t W_2^{i} \right\| =\left\| u(t_{i}) -u(t_{i-1})-\Delta t \frac{ u_{t}(t_{i})+ u_{t}(t_{i-1})}{2} \right\| \le C(\Delta t )^{2}\int _{t_{i-1}}^{t_{i}}\left\| u_{ttt}(t) \right\| dt , \end{aligned}$$

so that

22 $$\begin{aligned} (\Delta t )^{2} \sum _{i=1}^{k-1}\left\| W_2^{n-im}\right\| ^{2}\le C(\Delta t )^{4} \sum _{i=1}^{k-1}\Bigg (\int _{t_{n-im-1}}^{t_{n-im}}\left\| u_{ttt}(S) \right\| dt\Bigg )^{2} \le C(\Delta t )^{4}. \end{aligned}$$

From Eq. (21) and Eq. (22), we have

$$\begin{aligned} \left\| U^{n} -u (t_{n})\right\| \le C(h^{2}+(\Delta t)^{2}) ,for~~ n=1,2,\dots \end{aligned}$$

$$\square$$

## Numerical experiments

The performance of the proposed methods is tested by using numerical experiments. To evaluate errors, $L_{\infty }$ and $L_{2}$ error norms are applied as follows:

$$\begin{aligned} L_{\infty }= \mathop {\max }\limits _{1 \le n \le N } \left| {u(t_{n})-(U^{n})} \right| ,L_{2}=\sqrt{ h\sum _{i=1}^{N}\left| {u(t_{n})-(U^{n})} \right| ^{2}} \end{aligned}$$

Order of convergence is obtained by

$$\begin{aligned} Order =\frac{\log (E^{h_{1}}/E^{h_{2}})}{\log (h_{1}/h_{2})} \end{aligned}$$

where $E^{h_{1}}$ and $E^{h_{2}}$ represent the errors at step sizes $h_{1}$ and $h_{2}$, respectively.

### Example 1

[29] Consider

23 $$\begin{aligned} \left\{ \begin{array}{lll} \frac{\partial u(x,t)}{\partial t} = a_{1}\frac{\partial ^{2} u(x,t)}{\partial x^{2}}+a_{2}\frac{\partial ^{2} u(x,t-\tau )}{\partial x^{2}} ,t>0,0< x <\pi , \\ u(x,t) =\psi \ (x,t), ~~-\tau \le t\le 0,0 \le x \le \pi ,\\ u(0,t)=u(\pi ,t)=0, t>0. \end{array} \right. \end{aligned}$$

First, we take the initial function as $\psi (x,t) =sin(x),\tau =1,a_{1}=1.5,a_{2}=1$ such that the trivial solution of Eq.(1) is asymptotically stable. Numerical results are obtained and plotted at time $T=5$ using different $(\Delta t =\tau /m$,$h=\pi /N$).

**Fig. 1:**
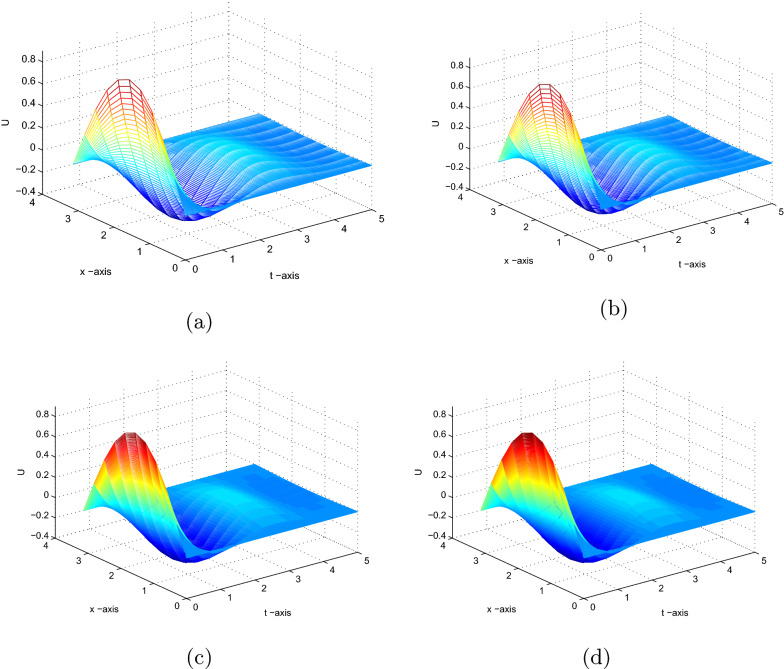
Solution of (23) with parameter values a) $N=10$ and $m=40$. b) $N=10$ and $m=50$. c) $N=10$ and $m=200$. d) $N=10$ and $m=500$

We apply the proposed method with different step sizes to solve the problem. The graph of numerical results is shown in Fig. 1. This graph shows that the numerical solution is asymptotically stable. And these confirm the theoretical results in Theorem 1.

### Example 2

[30] Consider

24 $$\begin{aligned} \left\{ \begin{array}{lll} \frac{\partial u(x,t)}{\partial t} = a_{1}\frac{\partial ^{2} u(x,t)}{\partial x^{2}}+a_{2}\frac{\partial ^{2} u(x,t-\tau )}{\partial x^{2}}+h(x,t),t>0,0< x <\pi , \\ u(x,t) =\psi \ (x,t), ~~-\tau \le t\le 0,0 \le x \le \pi , \\ u(0,t)=u(\pi ,t)=0, t>0, \end{array}\right. \end{aligned}$$

with the initial condition we take the initial function as $\psi (x,t) =\sin (x)$, and the added term *h*(*x*, *t*) where that is the exact solution is $u(x,t)=\exp ({-t})sin(x)$. Here, we take the parameters $a_{1}=1,a_{2}=0.5,\tau =0.5$ and compute the problem on $[0,\pi ]\times [0,2]$ for different space and temporal step sizes $(\Delta x=\pi /N,\Delta t=\tau /m)$.

**Table 1:** Errors norms and the corresponding convergence orders ($\Delta t\approx \Delta x^{2}$) for example 2

*N*	Central finite difference method ($\theta =1$) [30]				Linear B-spline FEM			
	$L_{2}$	Order	$L_{\infty }$	Order	$L_{2}$	Order	$L_{\infty }$	Order
5	5.41E−02	–	4.10E−02	–	2.52E−02	–	2.45E−02	–
10	1.34E−03	2.00	1.07E−02	2.07	4.77E−03	2.40	4.70E−03	2.38
20	3.25E−03	2.04	2.59E−03	2.02	1.14E−03	2.06	1.12E−03	2.07
40	8.10E−04	2.00	6.46E−04	2.00	2.83E−04	2.01	2.76E−04	2.02
80	2.02E−04	2.00	1.61E−04	2.00	7.06E−05	2.00	6.89E−05	2.00

**Table 2:** Comparison of the numerical solutions obtained with various values of *m* for $N = 10 , T = 1$, and $\tau =0.5$ with the exact solution for example 2

*x*	Numerical solutions				Exact solution
	*m* =10	*m* =20	*m* =40	*m* =80	
0.1$\pi$	0.187408	0.187423	0.187427	0.187427	0.187428
0.2$\pi$	0.356472	0.356500	0.356507	0.356509	0.356509
0.3$\pi$	0.490642	0.490680	0.490690	0.490692	0.490693
0.4$\pi$	0.576784	0.576829	0.576841	0.576843	0.576844
0.5$\pi$	0.606467	0.606514	0.606526	0.606529	0.606530
0.6$\pi$	0.576784	0.576829	0.576841	0.576843	0.576844
0.7$\pi$	0.490642	0.490680	0.490690	0.490692	0.490693
0.8$\pi$	0.356472	0.356500	0.356507	0.356509	0.356509
0.9$\pi$	0.187408	0.187423	0.187427	0.187427	0.187428

Table  1 shows the numerical errors and the corresponding orders. When the grid size is reduced, both error norms are significantly reduced. These results show the convergence of the linear B-spline finite element method. The given results suggest that the proposed method has order 2 of accuracy. The calculated error norms are also compared with the result obtained using the central difference method [30]. In Table  2, the comparison between the exact and approximation solution are given.

## Conclusion

In this paper, a finite element method is constructed based on linear B-spline basis functions for solving the generalized diffusion equations with delay. The detailed description of results through tables and graphs proves that the proposed numerical method is working efficiently. For all the test cases, simulations at a different set of data points are carried out to check the applicability of the numerical scheme. Based on these observations, our expectation that the given method is well suited to the generalized diffusion with the delay is confirmed.

## Limitations

The linear B-spline basis functions yields an order 2 of accuracy. One can use higher polynomial basis functions in order to increase the order of accuracy in space.

## Data Availability

No additional data is used for this research work.

## Abbreviations

FEM: Finite element method
PDEs: Partial differential equations
